# Assessment of hospital performance with a case-mix standardized mortality model using an existing administrative database in Japan

**DOI:** 10.1186/1472-6963-10-130

**Published:** 2010-05-19

**Authors:** Hiroaki Miyata, Hideki Hashimoto, Hiromasa Horiguchi, Kiyohide Fushimi, Shinya Matsuda

**Affiliations:** 1 Department of Healthcare Quality Assessment, Graduate School of Medicine, University of Tokyo, Japan; 2 Department of Health Economics and Epidemiology Research, School of Public Health, University of Tokyo, Japan; 3 Department of Health Management and Policy, Graduate School of Medicine, University of Tokyo, Japan; 4 Division of Healthcare Informatics, Department of Health Policy, Graduate School of Medicine and Dentistry, Tokyo Medical and Dental University, Tokyo, Japan; 5 Department of Preventive Medicine and Community Health, University of Occupational and Environmental Health, Japan

## Abstract

**Background:**

Few studies have examined whether risk adjustment is evenly applicable to hospitals with various characteristics and case-mix. In this study, we applied a generic prediction model to nationwide discharge data from hospitals with various characteristics.

**Method:**

We used standardized data of 1,878,767 discharged patients provided by 469 hospitals from July 1 to October 31, 2006. We generated and validated a case-mix in-hospital mortality prediction model using 50/50 split sample validation. We classified hospitals into two groups based on c-index value (hospitals with c-index ≥ 0.8; hospitals with c-index < 0.8) and examined differences in their characteristics.

**Results:**

The model demonstrated excellent discrimination as indicated by the high average c-index and small standard deviation (c-index = 0.88 ± 0.04). Expected mortality rate of each hospital was highly correlated with observed mortality rate (r = 0.693, p < 0.001). Among the studied hospitals, 446 (95%) had a c-index of ≥0.8 and were classified as the higher c-index group. A significantly higher proportion of hospitals in the lower c-index group were specialized hospitals and hospitals with convalescent wards.

**Conclusion:**

The model fits well to a group of hospitals with a wide variety of acute care events, though model fit is less satisfactory for specialized hospitals and those with convalescent wards. Further sophistication of the generic prediction model would be recommended to obtain optimal indices to region specific conditions.

## Background

Initiatives to measure healthcare quality attract serious attention from policy-makers and consumers who believe that such measurements can drive improvements in the quality of the service [1]. Recent enthusiasm for outcome evaluation such as in-hospital mortality, however, has been challenged because of the difficulties of ensuring adequate risk adjustment for different patient populations, an indispensable factor for fairly evaluating healthcare performance [2]. Owing to the clear definition of outcome and available knowledge on influential patient conditions, disease-specific risk adjustment models have been developed in several specialties, including cardiovascular diseases, and have been available for various quality improvement studies [3-6]. However, a risk adjustment model for a more generic use of outcome evaluation has not been fully developed [7]. In our previous study, we proposed and tested a generic risk prediction model to predict the risk of in-hospital mortality, with variables easily obtainable from large electronic administrative databases [8]. Our model showed excellent precision and calibration compared to other risk adjustment models [9-12].

However, the dataset used in the previous study was derived mainly from large university-affiliated teaching hospitals, which may compromise the ability to generalize results to a broader array of hospitals. Since the calculation of risk-adjusted in-hospital mortality is often conducted for benchmarking purposes, whether the risk adjustment model is applicable to hospitals with varying characteristics and case-mix must be clarified. To date, few studies have examined whether case-mix risk adjustment can be evenly applied such hospitals. In this study, we applied a generic case-mix-based risk adjustment model for in-hospital mortality prediction to hospitals with varying characteristics, and evaluated its performance for benchmarking risk-adjusted hospital mortality using a nationwide database of discharge cases.

## Methods

### Data source

We used an electronic, standardized dataset of discharged patients provided by 469 hospitals that participated in a Japanese patient classification system and related evaluation scheme from July 1 to October 31, 2006. The patient classification system, or Diagnosis Procedure Combination (DPC), includes information for up to two major diagnoses and up to six co-existing diagnoses. The 2008 version of the DPC system includes 18 major diagnostic categories (MDC) and 506 disease subcategories coded in ICD10. For analytic purposes, we re-categorized the 18 MDCs into 10 MDCs based on mortality rates. The dataset also includes additional information such as patient demographics, uses and types of surgical procedures, emergency/elective hospitalization, length of stay, and discharge status (including in-hospital death) [13-15]. Records for 1,878,767 discharge cases were available for the following analysis. Cases were randomly assigned into two subsets with an approximate 50/50 split: one for model development and the other for validation tests. The obtained model development dataset included 939,409 records and the validation dataset included 939,358 records. Because of the anonymous nature of the data, the requirement for informed consent was waived. Study approval was obtained from the institutional review board of the hospital with which the last author was affiliated.

### Model building and validation

We started with the mortality prediction model used in our previous study [8]. The model includes age, gender, use of an ambulance at admission, admission status (emergency/elective), MDC of the primary diagnosis, and comorbidity. Based on Quan's methodology [9], the ICD-10 code of each co-existing diagnosis was converted into a Charlson Comorbidity Index score. We classified scores into five categories: 0, 1-2, 3-6, 7-12, and 13 and over. We further modified our former model by including "admission purpose." In the previous study, we found that the mortality risk of patients with cardiovascular diseases tended to be underestimated because this group of patients included those hospitalized only for post-operative evaluation. Thus, including admission purpose should improve the precision of low-risk prediction. We also included Eastern Cooperative Oncology Group performance status (grade 0, fully active; grade 4, completely disabled) [16] and Fletcher-Hugh-Jones classification of respiratory status (class 1, patient's breathing is similar to others of the same sex and age; class 5, patient is breathless when talking or undressing, or is unable to leave the house due to breathlessness) [17]. These parameters were included because the mortality risk of patients with cancer and chronic pulmonary diseases tended to be overestimated, and inclusion of these additional scores should improve predictive precision for such patients. Given that Fletcher-Hugh-Jones classification and performance status scores were required only for those with chronic pulmonary diseases and cancer, missing observations were treated as null values. A multivariate logistic regression analysis including variables mentioned above was performed to predict in-hospital mortality using the development dataset. The tests of model performance and fitness were conducted using the test dataset. Accuracy of the prediction models was determined with the c-index [18]. We assessed the ability of the model to accurately predict mortality across all ranges of risk by comparing predicted and observed mortality rates in predicted mortality risk deciles.

### Comparison of hospital performance

We excluded from analysis one hospital that had a mortality rate of zero because the c-index could not be calculated. Given that a c-index of 0.8 to 0.9 is considered excellent [19], we divided hospitals into two groups by setting a c-index of 0.8 as the cut-off point. We then examined differences in characteristics between the two groups of hospitals, including size, number of admissions, crude and predicted mortality, and distribution of patient demographics and diseases using Fisher's exact test and the t-test as appropriate. Hospitals for which the sole MDC category accounted for more than half of all hospitalized cases were considered "specialized hospitals." All statistical tests were 2-tailed and the significance level was set at p < 0.05.

Standardized mortality ratios (SMRs) were obtained by calculating the ratio of observed mortality to expected mortality estimated by the model. Standardized mortality rate was obtained by multiplying SMRs and the average in-hospital mortality rate for all hospitals. All analyses were conducted with SPSS version 15.0J (SPSS Japan, Inc).

## Results

Table 1 shows patient characteristics in the development and validation datasets. Among the 939,409 patients (male, 53.0%; age under 50 years at admission, 32.6%; age of 90 years or older, 1.6%) in the development dataset, the MDC with the highest proportion was the "digestive system (20.7%)," followed by "skin, ear, eye, pediatric, and newborn (14.7%)," "musculoskeletal, injuries, and others (14.6%)," "respiratory system (10.7%)," "circulatory system (9.8%)," "female, breast (8.8%)," "kidney (7.8%)," "nervous system (6.9%)," "endocrine (3.4%)," and "blood, blood forming organs, and immunological disorders (2.4%)." The majority of patients (69.5%) had a total score of 0 for the Charlson Comorbidity Index, and only 2.5% of patients had a score higher than 6. With regard to admission status, 42.3% had emergency status, 12.5% used an ambulance, 6.7% stayed in the hospital for examination, and 4.7% planned short-term admissions. For cancer performance status, almost all patients were grade 0, grade 1, or missing (98.1%), while only 1.8% of patients were grade 2 or higher. For the Fletcher Hugh-Jones classification, almost all patients were class 1, class 2, or missing (97.1%), while only 2.9% of patients were class 3 or higher.

**Table 1:** Patients characteristics in development dataset (n = 939,409) and validation dataset (n = 939358)

		Development Dataset		Validation Dataset	
		N	%	N	%
Major Diagnostic Category	Digestive system	194,257	20.7	193,857	20.6
	Respiratory System	100,591	10.7	101,001	10.8
	Blood and Blood Forming Organs and Immunological Disorders	22,741	2.4	23,009	2.4
	Kidney	73,240	7.8	73,353	7.8
	Nervous System	65,096	6.9	64,619	6.9
	Circulatory System	91,892	9.8	92,188	9.8
	Female, Breast	82,732	8.8	82,696	8.8
	Endocrine	31,528	3.4	31,488	3.4
	Skin, Ear, Eye, Pediatric and Newborn	139,642	14.7	139,614	14.9
	Musculoskeletal, injuries and others	137,690	14.7	137,533	14.6

Sex	Male	497,564	53.0	307,974	32.8

Age (years)	under50	306,473	32.6	134,255	14.3
	50-59	134,905	14.4	185,643	19.8
	60-69	186,102	19.8	207,560	22.1
	70-79	208,171	22.2	89,130	9.5
	80-89	88,922	9.5	14,796	1.6
	90 and over	14,836	1.6	307,974	32.8

Total score of Charlson Index	score0	652,986	69.5	651,578	69.4
	score1,2	209,063	22.3	209,543	22.3
	score3-6	53,781	5.7	53,974	5.7
	score7-12	17,645	1.9	18,202	1.9
	score13-	5,934	0.6	6,061	0.6

Admission Status	Status emergency	397,650	42.3	397,802	42.3
	Use of an ambulance	116,987	12.5	116,988	12.5
	Hospitalization for examination	62,673	6.7	63,012	6.7
	Planned short-term admission	44,434	4.7	44,494	4.7

Performance Status	grade0, grade1, or missing	921,721	98.1	921,533	98.1
	grade2	7,544	0.8	7,724	0.8
	grade3	5,103	0.5	5,103	0.5
	grade4	5,041	0.5	4,998	0.5

Fletcher Hugh-Jones Classification	class1, class2, or missing	912,266	97.1	911,891	97.1
	class3, class4	16,082	1.7	16,306	1.7
	class5	110,61	1.2	11,161	1.2

In-hospital	Mortality	34,636	3.7	34,866	3.7

C-index		0.882		0.882	

Table 2 shows the in-hospital mortality prediction model applied to the development dataset. Using the "musculoskeletal, injuries, and others" MDC as a reference, MDCs for "endocrine" and "skin, ear, eye, pediatric, and newborn," showed a significantly lower odds ratio for in-hospital deaths compared to other MDCs. Older age, male gender, use of ambulance at admission, and emergency admission status showed a significantly higher odds ratio. Hospitalization for examination and planned short-term admission showed a significantly lower odds ratio. As scores increased for the Charlson Comorbidity Index, performance status, and Fletcher-Hugh-Jones classification, the odds ratio exhibited a linearly increasing trend.

**Table 2:** Detail of mortality prediction model of development dataset (n = 939,409)

		odds ratio	95% CI	
			lower	upper
Major Diagnostic	Digestive system	1.81	1.73	1.89
Category	Respiratory System	1.56	1.48	1.65
	Blood and Blood Forming Organs and Immunological Disorders	5.49	5.16	5.84
	Kidney	1.40	1.32	1.49
	Nervous System	1.80	1.71	1.89
	Circulatory System	1.76	1.67	1.85
	Female, Breast	1.16	1.07	1.27
	Endocrine	0.62	0.56	0.69
	Skin, Ear, Eye, Pediatric and Newborn	0.39	0.35	0.42
	Musculoskeletal, injuries and others	1.00	(reference)	

Sex	Male	1.23	1.20	1.26

Age (years)	under50, 50-59, 60-69,70-79, 80-89, 90 and over	1.45	1.44	1.46

Total score of Charlson Index	score0	1.00	(reference)	
	score1,2	1.23	1.20	1.27
	score3-6	3.17	3.05	3.29
	score7-12	5.92	5.61	6.26
	score13-	10.30	9.51	11.14

Admission Status	Status emergency	2.80	2.71	2.89
	Use of an ambulance	2.39	2.33	2.46
	Hospitalization for examination	0.11	0.09	0.13
	Planned short-term admission	0.25	0.23	0.28

Performance Status	grade0, grade1, or missing	1.00	(reference)	
	grade2	3.00	2.79	3.23
	grade3	7.29	6.81	7.81
	grade4	23.98	22.35	25.73

Fletcher Hugh-Jones Classification	class1, class2, or missing	1.00	(reference)	
	class3, class4	1.47	1.38	1.57
	class5	5.26	4.97	5.57

**Figure 1:**
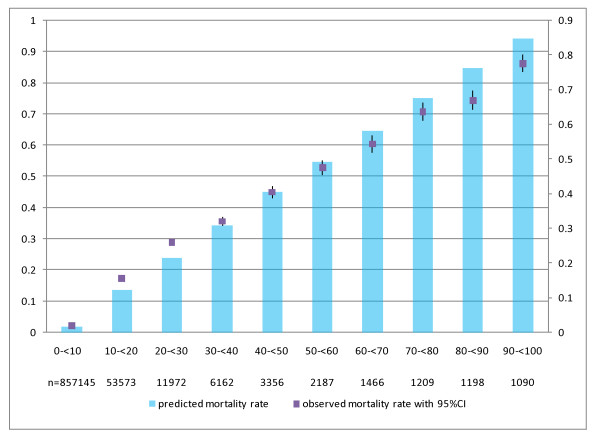
**Predicted and observed mortality by risk decile (n = 939,358)**. The horizontal axis shows ten predicted mortality ranges. The total number of patients in these ranges is shown in the lower columns. The observed mortality rate with its associated 95% confidence interval is shown by the dark square. The predicted mortality rate is indicated by bar graphs. The c-index of the model is 0.882.

The risk prediction model exhibited a c-index of 0.882 for both development and validation datasets. Predicted and observed deaths in the validation dataset are shown in Figure 1 by risk decile. Expected mortality was lower than observed mortality in higher deciles, whereas the reverse was observed in lower deciles.

**Figure 2:**
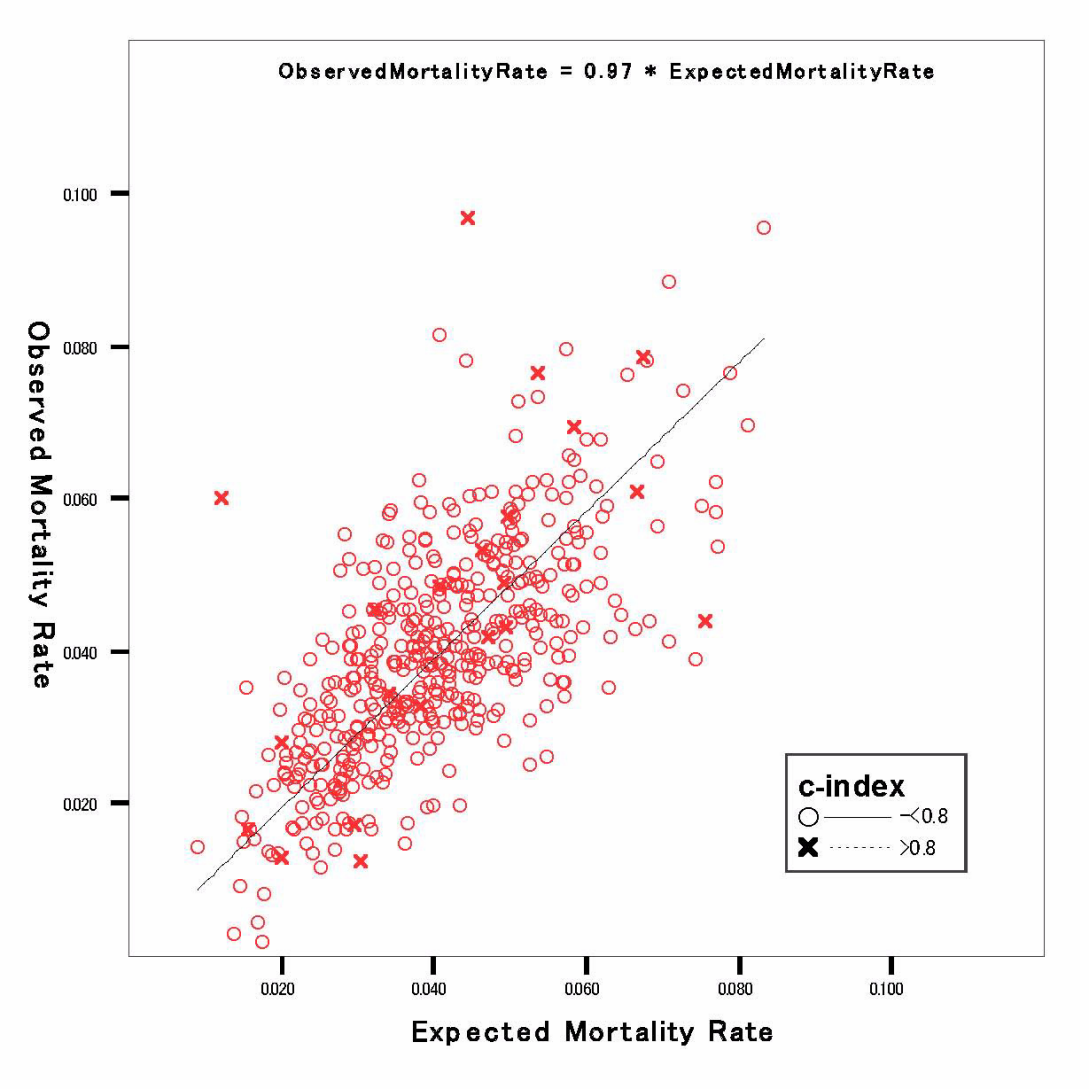
Expected versus observed hospital mortality rate (n = 468*). Each dot represents data from one hospital (r = 0.693, p < 0.001). Expected mortality rates represent average predicted risk in each hospital. *One hospital (mortality rate = 0%) was excluded from this analysis.

Table 3 summarizes major characteristics of the 468 hospitals (mean ± standard deviation of c-index for each hospital, 0.88 ± 0.04). Among these hospitals, 446 were allocated to the higher c-index group (average c-index; 0.882, 95%CI; 0.878-0.885), and 22 to the lower c-index group (average c-index; 0.772, 95%CI; 0.757-0.786). The higher c-index group had a significantly higher number of admissions, hospital mortality rate, and standardized mortality rate. Hospitals in the lower c-index group were significantly more likely to be specialized hospitals, hospitals with convalescent wards, and private hospitals. Figure 2 plots expected and observed mortality by higher and lower c-index groups (expected mortality rates represent average predicted risk in each hospital). The lower c-index group tended to be positioned off-diagonal in the plot, but no systematic trend of overestimation or underestimation was found between the two groups. Expected mortality in each hospital was highly correlated with observed mortality (total, r = 0.693, p < 0.001). The correlation between expected and observed mortality in the higher c-index group (r = 0.702, p < 0.001) was higher compared to that of the lower c-index group (r = 0.663, p < 0.01). The average observed mortality to expected mortality (OE) ratio by risk decile for hospitals is shown in Table 4. A comparison of the standardized and raw mortality rate quartiles is displayed in Table 5. After risk adjustment, 62% percent of hospitals (n = 290) were categorized in a different quartile.

**Table 3:** Characteristics of hospitals (n = 468*)

		All	Hospitals	c-index	->0.8	c-index	< 0.8	P value
			N = 468		N = 446		N = 22	
		Mean	SD	Mean	SD	Mean	SD	
C-index		0.88	0.04	0.88	0.03	0.77	0.03	<0.001

Number of admission		4011.5	2539.4	4128.9	2528.6	1631.7	1297.3	<0.001

Hospital mortality rate		4.0%	1.5%	4.0%	1.4%	5.0%	2.9%	0.001

Average predicted risk		4.0%	1.3%	4.0%	1.3%	4.4%	1.9%	0.251

Standardized mortality rate		3.8%	1.3%	3.7%	1.1%	4.7%	3.4%	0.001

Major Diagnostic Category	Digestive system	21.2%	8.5%	21.6%	8.2%	14.7%	11.7%	<0.001
	Respiratory System	11.0%	4.5%	11.1%	4.4%	9.5%	6.7%	0.106
	Blood and Blood Forming Organs and Immunological Disorders	2.0%	1.6%	2.1%	1.6%	1.1%	1.2%	0.007
	Kidney	7.9%	5.5%	7.8%	4.8%	8.9%	13.4%	0.351
	Nervous System	7.8%	8.4%	7.2%	6.6%	20.1%	22.2%	<0.001
	Circulatory System	10.0%	9.2%	9.8%	8.3%	14.1%	20.2%	0.033
	Female, Breast	7.5%	5.9%	7.7%	5.8%	2.4%	6.4%	<0.001
	Endocrine	3.3%	1.7%	3.4%	1.7%	2.9%	2.0%	0.224
	Skin, Ear, Eye, Pediatric and Newborn	13.6%	5.9%	13.9%	5.8%	6.9%	5.0%	<0.001
	Musculoskeletal, injuries and others	15.6%	8.7%	15.5%	8.3%	19.2%	15.4%	0.052

Sex	Male	52.8%	4.8%	52.8%	4.7%	54.6%	6.8%	0.072

Age (years)	under50	30.4%	9.9%	30.9%	9.6%	19.5%	9.5%	<0.001
	50-59	13.8%	2.8%	13.8%	2.7%	13.9%	3.7%	0.878
	60-69	19.4%	3.6%	19.4%	3.5%	20.1%	5.1%	0.346
	70-79	23.0%	4.3%	22.8%	4.2%	27.0%	4.4%	<0.001
	80-89	11.3%	4.6%	11.0%	4.3%	16.2%	7.1%	<0.001
	90-	2.1%	1.6%	2.1%	1.5%	3.4%	2.5%	<0.001

Admission Status	Status emergency	47.0%	15.6%	46.7%	15.2%	51.6%	22.0%	0.155
	Use of an ambulance	13.6%	7.3%	13.4%	6.8%	19.3%	12.6%	<0.001
	Hospitalization for examination	4.0%	5.8%	4.1%	5.9%	3.0%	5.2%	0.374
	Planned short-term admission	6.8%	5.9%	6.6%	4.7%	11.0%	16.9%	0.001

		N	%	N	%	N	%	P value

Public hospital**		130	27.8	128	28.7%	2	9.1%	0.025
University hospital**		77	16.5%	76	17.0%	1	4.5%	0.079
Special hospital**		25	5.3%	18	4.0%	7	31.8%	<0.001
Have convalescent wards**		43	10.0%	38	9.3%	5	26.3%	0.025

*One hospital (mortality rate = 0%) was excluded from this analysis

**percentage of total hospitals

**Table 4:** Hospital average OE ratio** by risk deciles (n = 468)

Hospital average preoperative risk	Number of hospitals	Average OE ratio	95% CI	
			lower	upper
Under 2.0%	21	1.15	0.68	1.62
2.0-2.5%	34	1.11	1.01	1.21
2.5-3.0%	61	1.05	0.96	1.14
3.0-3.5%	59	1.11	1.03	1.19
3.5-4.0%	67	1.02	0.96	1.09
4.0-4.5%	68	1.03	0.96	1.10
4.5-5.0%	49	0.95	0.89	1.00
5.0-5.5%	44	0.96	0.88	1.03
5.5-6.0%	33	0.88	0.82	0.95
6.0% and over	32	0.89	0.80	0.98

**OE ratios means "Observed mortality/Expected mortality ratio"

**Table 5:** Hospital standardized mortality rates quartile by raw mortality rates quartile (n = 468*)

		Standardized Mortality Rates percentile				
		-25^th ^(-<3.02%)	25th-50th (3.02%-<3.60%)	50th-75th (3.60%-<4.33%)	75th-(4.33%>)	Total
Raw Mortality Rates percentile	-25th (-<3.02%)	57	26	21	13	117
	25th-50th (3.02%-<3.89%)	34	39	19	25	117
	50th-75th (3.89%-<4.90%)	21	31	34	31	117
	75th-(4.90%>)	5	21	43	48	117

	total	117	117	117	117	468

* One hospital (mortality rate = 0%) was excluded from this analysis

## Discussion

In this study, we developed a modified case-mix-based risk adjustment model for in-hospital mortality using administrative data, and tested its performance in various types of hospitals. The model demonstrated excellent discrimination as indicated by the high average c-index, and was applicable to the majority of hospitals in our sample set taken from a large hospital discharge database. However, our finding that a few hospitals had a lower c-index warrants further discussion.

The hospitals with a lower c-index were characterized by a case-mix predominantly involving circulatory and nervous system disorders, and older patients with higher mortality. These characteristics indicate that hospitals with a lower c-index were those that provided a combination of acute and long-term care. As is often reported, Japanese hospitals, especially small/middle-sized private hospitals, are not well differentiated with respect to provision of acute and long-term care [20]. The hospitals with a lower c-index provided both acute and long-term care specifically to stroke patients. Although the Japanese patient classification system includes the majority of acute-care hospitals, and our dataset should cover a large share of these hospitals, the recent expansion of the system to include a wider range of hospitals has led to increased heterogeneity in the functions of participating hospitals. Our results may suggest that the proposed risk prediction model does not apply as well to mixed-care hospitals, and should be selectively applied to general hospitals that provide acute care.

Our model demonstrated excellent discrimination without the need for detailed clinical data. As discussed in a previous study [8], our model's high predictive precision was made possible by including patient demographics and admission status, further combined with MDCs and the Charlson Comorbidity Index. All variables are easily accessible from administrative data properly coded with internationally standardized disease codes such as ICD-10, and allows for excellent model performance. Our model framework may be applicable and useful in other countries as well.

Public disclosure of hospital performance (e.g., hospital-standardized mortality rate) is considered to provide informed choice to consumers/patients, provide a benchmark for hospital management, and enhance efficiency of the health care system by stressing competition over quality. Proper risk adjustment then becomes crucial for providing unbiased information on the quality of hospital performance. As we have demonstrated, risk adjustment had a marked impact on hospital ranking, since a larger share of hospitals shifted to a different quartile of hospital mortality rate after adjustment. These results suggest that our model can be used for benchmarking hospital-standardized mortality rate with fair risk adjustment among acute-care hospitals.

A potential limitation of our study worth noting is the quality of diagnosis coding in the database. We relied on original data submitted by participating hospitals, simply because the same information is used in actual billing statements for claim reimbursement. Our preliminary analysis did not identify serious flaws in the quality of ICD10 codes, although the quality of coding and how it affects the precision of risk prediction may be an important issue to be addressed in future studies. Regional applicability, however, may be more of a concern for the risk adjustment framework. A recent international comparative study [21] demonstrated that while cross-national application of a formula can achieve high predictive accuracy, the level of accuracy varied across countries [8,21]. This may be partly because disease distribution and burden are different between countries with different health care systems. Thus, it may be preferable for investigators to develop "optimal" indices for their own data-specific and condition-specific model coefficients.

Physicians and hospitals will strongly oppose public reporting if risk-adjusted outcomes are not reflective of provider-specific performance [22]. Enhanced validity and reliability of standardized mortality rates and other risk-adjusted outcomes may be essential not only for benchmarking, but also for public reporting. Utilizing process measures in conjunction with risk-adjusted outcomes may also be used for quality improvement, as some research has documented an association between higher adherence to care guidelines and better outcomes of patients who receive that care [23,24]. However, other research has suggested that hospital performance measures predict small differences in hospital risk-adjusted mortality rates [25]. Further efforts are needed to develop performance measures that are tightly linked to patient outcomes. We also note that in-hospital mortality reflects just one aspect of hospital performance. In order to properly reflect patient values, it may be necessary to assess hospital performance using other factors as well, such as potentially avoidable adverse events (e.g., readmission and complications) [26].

## Conclusion

The risk model developed in this study exhibited a good degree of predictive accuracy for benchmarking hospital mortality with variables easily accessible from administrative data. The model fits better to and can be applied selectively to benchmarking general acute care hospitals. However, model fit is less satisfactory for specialized hospitals and those with convalescent wards. Further sophistication of the generic prediction model would be recommended to obtain optimal indices to region specific conditions.

## Competing interests

The authors declare that they have no competing interests.

## Authors' contributions

HM conceived the study and designed the protocol. HM and HH1 wrote the manuscript. HH2, KF, and SM managed data collection and processing. All authors have read and approved the final manuscript.

## Pre-publication history

The pre-publication history for this paper can be accessed here:

http://www.biomedcentral.com/1472-6963/10/130/prepub
